# Phenotypic screening of low molecular weight compounds is rich ground for repurposed, on-target drugs

**DOI:** 10.3389/fphar.2022.917968

**Published:** 2022-08-08

**Authors:** Christopher A. Lipinski, Andrew G. Reaume

**Affiliations:** Melior Discovery, Exton, PA, United States

**Keywords:** phenotypic screening, drug repositioning, low molecular weight drugs, rule-of-five, MLR-1023, drug discovery, medicinal chemistry, pleiotropy

## Abstract

A target-based drug discovery strategy has led to a bias away from low molecular weight (MWT) drug discovery. Analysis of the ACS chemistry registration system shows that most low MWT drugs were first made in the time era before target-based drug discovery. Therapeutic activity among most low MWT drugs was identified in the era of phenotypic drug discovery when drugs were selected based on their phenotypic effects and before *in vitro* screening, mechanism of action considerations and experiences with fragment screening became known. The common perception that drugs cannot be found among low MWT compounds is incorrect based on both drug discovery history and our own experience with MLR-1023. The greater proportion of low MWT compounds that are commercially available compared to higher MWT compounds is a factor that should facilitate biology study. We posit that low MWT compounds are more suited to identification of new therapeutic activity using phenotypic screens provided that the phenotypic screening method has enough screening capacity. On-target and off-target therapeutic activities are discussed from both a chemistry and biology perspective because of a concern that either phenotypic or low MWT drug discovery might bias towards promiscuous compounds that combine on-target and off-target effects. Among ideal drug repositioning candidates (late-stage pre-clinical or clinically-experience compounds), pleiotropic activity (multiple therapeutic actions) is far more likely due to on-target effects arising where a single target mediates multiple therapeutic benefits, a desirable outcome for drug development purposes compared to the off-target alternative. Our exemplar of a low MWT compound, MLR-1023, discovered by phenotypic screening and subsequently found to have a single mechanism of action would have been overlooked based on current era medicinal chemistry precedent. The diverse therapeutic activities described for this compound by us, and others arise from the same pleiotropic lyn kinase activation molecular target. MLR-1023 serves as a proof-of-principle that potent, on target, low MWT drugs can be discovered by phenotypic screening.

## Today’s small molecule drug discovery is biased towards MWT 300–500

Broadly speaking all small molecule drugs in existence today are the product of either a pre-target-based discovery era or post-target-based discovery era. Since the mid-1970s the practice of screening chemical libraries using high throughput target-based screens has favored drug candidates with higher binding affinity, higher specificity, and by extension, higher molecular weight (MWT).

**FIGURE 1:**
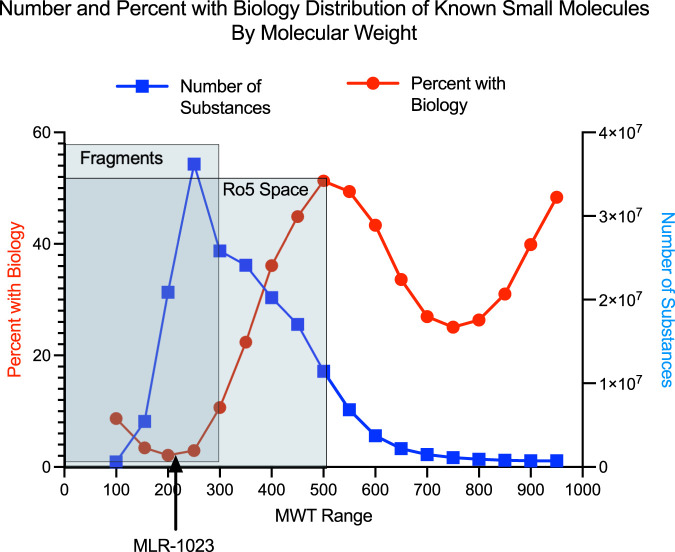
Number of compounds in each 50 MWT range interval is shown with the blue line. The percent of compounds in each 50 MWT range which have a reported biology study associated with them is shown with the red line. The American Chemical Society Chemical Abstracts Service (CAS) has been abstracting the chemistry literature for over 100 years (Schenck and Zapiecki, 2014). Currently both chemistry publications and many biological publications having at least some chemistry content are abstracted and any chemical compounds found in either chemistry or biology publications are either indexed under an existing CAS registry number for a previously reported compound or are assigned a new unique CAS registry number. A variety of experimental, computational and annotated descriptors are associated with almost all CAS registry numbers. Examples of exceptions occur for the case of incompletely structurally defined compounds, for mixtures or for compounds whose structure is for some reason indeterminate. The CAS coverage of patents having chemistry content is very complete. Patents from every patent issuing country across the world are abstracted and all non-English patents are translated into English. A chemistry feature found in patents and virtually never in peer reviewed journals is the presence of “prophetic compounds” (CAS, 2022a). “CAS Coverage of Prophetic Substances, ”(CAS.org: American Chemical Society). It is common in chemistry related patents to find lists of chemical compounds identified by chemical name but without any experimental evidence of synthesis and with some type of biological activity claimed for compounds in the list. Such compounds are referred to as “prophetic compounds” and are abstracted by CAS and are associated with an existing CAS registry number for previously abstracted compounds or are given new CAS registry numbers for newly described compounds. A reader of this type of patent can infer that a listed prophetic compound is viewed by the patent inventors as very likely to possess the stated type of biological activity but without much or any indication of the exact potency of the compound. In our experience, one can reliably conclude that a chemical compound is novel, if a chemical structure is searched against the CAS registry database and the search fails to find a CAS registry number. The CAS registry database is proprietary to the American Chemical Society. Detailed comparison between public and proprietary chemistry databases is found in a 2015 Journal of Medicinal Chemistry miniperspective article (Lipinski et al., 2015). Using SciFinder-n it is possible to determine the relationship between molecular weight range and the presence of some type of “biological study”. CAS defines the term “biological study” as a super role consisting of 24 subsets of more narrowly defined roles (CAS, 2022b). “Super Roles and Roles” (CAS.org: American Chemical Society). The reader can determine the definitions of the 24 roles by accessing the hyperlinked definitions in the Super Roles and Roles web page reference.

We performed the analysis depicted in Figure 1 which compares the relationship between MWT distribution and the presence of “biological study” for a compound among 184,139,678 chemical compounds in the American Chemical Society CAS registry system. The analysis illustrates that amongst all 184 million unique CAS registered molecules between MWT 100 and 999, the vast majority of those that have been linked to a biological study are greater than 300 MWT.

The search strategy employed in the SciFinder analysis was as follows: 184,139,678 chemical structures with MWT between 100 and 999 were found as of March 18, 2022. Searches on MWT range increments of 50 units of MWT were performed between MWT 100 and 999. For each MWT range of 50 the number of compounds was recorded as well as the number of compounds having the SciFinder-n super role of “biological study”. The search gave eighteen MWT ranges between MWT 100 and MWT 999. For each of these MWT ranges the fraction of compounds with the super role of “biological study” was determined. The advantage of this type of approach is that it answers the global question of the relationship of MWT range to the presence of some type of “biological study” as defined by CAS on an extremely large dataset of very diverse chemical compounds over a very broad collection of literature sources. To the authors knowledge this is the first reported description of this type of analysis. This analysis complements and agrees with published literature analyses on smaller sets of chemical compounds over more narrow ranges of biological study but with an expert pharmaceutical perspective (Leach and Hann, 2011). Briefly, authors at GSK described a theoretical model as to why one would expect that as chemical compounds become larger the binding mode possibilities to a target become smaller. The theoretical model was found to be consistent with experimental GSK biology screening data. Complementing earlier work, the advantage to our study is twofold. The number of compounds in each MWT range is in the millions and the MWT range varies considerably allowing a robust assessment of the relationship of MWT to biological activity across a huge chemical database (see Figure 1).

The nine MWT ranges between 150 and 599 contain 167,922,426 compounds and each MWT range contains more than five million compounds. The MWT range between 150 and 599 encompasses, in part, the regions of chemical MWT space covered by chemical compounds referred to as fragments (Erlanson et al., 2016) with MWT’s below 300. Such compounds typically have low affinity, in the 100 micro-molar to low milli-molar range in biological assays, and usually are detected using assay techniques capable of measuring very low affinity binding. Chemical compounds referred to as “rule of 5” compounds having MWT’s above 300 and below 500 were the predominant small molecule, non-natural products, found in drug discovery through the end of the twentieth century and are still the easiest to discover and develop through the current time (Lipinski, 2016).

Below MWT 150 and above MWT 600 the numbers of compounds in each MWT range are fewer than five million. The chemical structures of the higher MWT compounds with an increasing incidence of biological study are dominated by a few chemical classes such as peptides that are likely published and abstracted because these types of compounds are primarily of biological interest.

This current analysis based on the relationship of MWT range to the presence of some type of “biological study” as defined by CAS on an extremely large dataset of very diverse chemical compounds over a very broad collection of literature sources complements and considerably extends our previous observations based on the fragment literature. It is reflective of conventional thinking amongst medicinal chemists that compounds in the low MWT fragment range are predicted to have low binding affinity and likely would not be detected in the typical 1 to 10 micro-molar biochemical assay screening range. This conventional thinking argues that such compounds might not exhibit good specificity and thereby might be described as promiscuous.

## There is strong precedent for drug-like compounds in the very low MWT range

**FIGURE 2:**
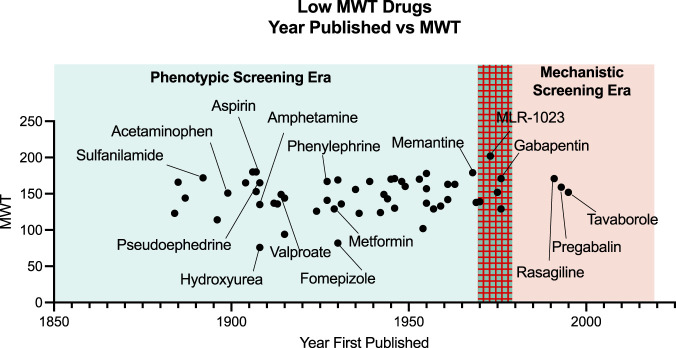
Fiftysix drugs referenced by Derek LoweLowe, D. (2014). The smallest drugs. (Online). Available: https://www.science.org/content/blog-post/smallest-drugs (Accessed March 21, 2022). And the year that they were published are plotted as Year First Published *vs*. MWT of the drug. Prior the 1970s all drugs were screened by some form of phenotypic screen as opposed to *in vitro* target based screening methods that arose in the 1970s.

Contrary to the view just depicted on the relationship of MWT to biological study, there in fact are many marketed drugs with MWT below 300. In 2014 the very experienced medicinal chemist Derek Lowe, in a blog discussion about very low MWT drugs, compiled a list of all drugs below MWT 180. From his list we have determined the date of the first literature disclosure of the chemical structure for each drug. The names of the drugs with MWT of 180 or less were searched in the ACS CAS SciFinder-n software for the earliest literature reference to the chemical structure. The results (Figure 2) clearly show that the chemical structure of most very small drugs was disclosed before the era of mechanistic drug screening began in the mid 1970s. In some cases, there was a long delay between the first disclosure of the chemistry structure and the disclosure of biological utility. Therefore, most of these low MWT drugs with MWT 180 or less were not discovered by reductionist mechanistic screening but rather by the phenotypic screening or clinical observations that preceded the onset of mechanism-based target screening.

In SciFinder-n, searching the range of MWT 76 to 180 we found 2.7 million compounds. Among the 2.7 million compounds, 2 million (75%) were commercially available. Having a compound that is commercially available is, at least in the initial stages of investigation, a big advantage to a biology investigator in that the compound does not have to be synthesized. Among these, 2 million low MWT commercially available compounds 5.9% were associated with biological study^1^.

By comparison, a search within the “rule of 5” MWT range of 400–500 found 38 million compounds. Among the 38 million compounds, 31% were commercially available with 5.5% of those *commercially* available reported to have some type of biological activity^2^ (in contrast to 40.1% with biological activity amongst *all* in this MWT range). The implications are that to a biology investigator looking for therapeutic candidate starting points the low MWT range is more commercially available (75 *vs*. 31% commercial availability) than the higher MWT range. The density of commercially available compounds with known biology is quite similar between low and high MWT compounds (5.5% for low MWT *vs*. 5.9% for higher MWT).

Many compounds in the very low MWT range will have been described that were originally not intended for biological study (e.g. for use as chemical intermediates). A perusal of the Derek Lowe small drugs list shows a number of compounds that are capable of covalent chemistry (e.g. cytotoxic cancer chemotherapy compounds). The medicinal chemistry viewpoint regarding the desirability of covalent chemistry in drugs has varied over time. An unfavorable viewpoint dominated 20 years ago. Currently the view on covalent acting drugs is much more balanced as summarized in a recent review article (De Vita, 2020).

The “biological study” density of compounds in chemical space is known to be very dependent on MWT with far fewer compounds existing at lower MWT ranges than at higher. As a rule, screening libraries of low MWT compounds do a better job of searching available chemistry space for biological activity than do libraries of higher MWT compounds (Hesterkamp and Whittaker, 2008). The tradeoff is that biological potency is generally weaker at low MWT than at high MWT. This tradeoff has discouraged researchers from screening low MWT compounds at typical HTS screening concentrations in the 1 to 10 micro-molar range. However low MWT drugs really do exist as pointed out in the discussion by Derek Lowe where he made the point with one exemplar, metformin: “Metformin alone is a constant rebuke to a lot of our med-chem prejudices: who among us, had we never heard of it, would not have crossed it off our lists of screening hits? So give these small things a chance, and keep an open mind. They’re real, and they can really be drugs” (Lowe, 2014).

We posit that the chemical space of compounds below MWT 300 may be more fruitful grounds for drug candidates than conventional wisdom has it and indeed fruitful grounds for identifying drug repositioning candidates. But, given that these are very low MWT compounds the question that we want to turn to is: When new indications for drugs in this space are found is it because the drugs are promiscuous, modulating more than one target because as very low MWT compounds target specificity is poor, or is there an alternative explanation?

## Evidence that most newly identified indications are on-target

As a company with a core activity of using *in vivo* phenotypic screening to uncover new therapeutic indications among drug repositioning candidates, it has been our observation that about one third of the over three hundred compounds screened by Melior in a panel of animal models (*thera*Trace^®^) show therapeutic activity that was not otherwise predicted. Moreover, it is our further observation that in those scenarios where we can determine, be it circumstantially or definitely, a mechanism for the newly identified activity, about ninety percent of the time the therapeutic target associated with the new activity appears to be the same target associated with the original activity. Compounds screened by Melior are not low information content compounds. Rather these compounds are high value where the sponsor has reduced the probability of toxicity and has enhanced the desired activity through chemistry and biology optimization. By way of emphasis, the “screening” set of compounds that we are referring to is not a random chemical substrate but rather is a set of ideal repositioning candidates. At least half of these compounds had clinical data and the balance were clinical candidates. Therefore, in all cases compounds would have been medicinal chemistry optimized to some extent thereby biassing them towards target selectivity. The implication of this observation is that most of the unexpected biological activity discovered by broad-based phenotypic activity screening on high value compounds is on-target rather than off-target. From a medicinal chemistry perspective most of the compounds being studied are in a pejorative language sense not promiscuous or in a positive language sense are not multi-target drugs.

## Use of the term pleiotropy as applied to drug repositioning

Broadly speaking, instances where a drug which exhibits multiple, independent therapeutic benefits has been referred to as pleiotropy or perhaps more accurately pharmacological pleiotropy, a derivation of pleiotropy used in a genetic sense which refers to the phenomenon where one gene affects multiple traits (O'Mara et al., 2019). A chemical candidate for drug repurposing can be a pleiotropic drug in the classic genetic sense in that the old indication and newly found repurposing discovery comes from involvement of a single gene product/target that exhibits different phenotypic/therapeutic effects. This type of drug repurposing is sometimes referred to as on-target (Childers et al., 2020).

Another type of drug repurposing candidate refers to a “pleiotropic” drug in which the “pleiotropic” annotation is not that of the classical genetic pleiotropy definition but rather comes from a looser terminology referring to a drug being associated with multiple phenotypic effects independent of whether those effects are associated with a single or multiple targets. The statins are an example where this type of looser terminology has been used (Yu and Liao, 2022). Multiple (pleiotropic) effects arising from a drug acting on multiple targets unrelated to any common gene are not uncommon with estimates in the medicinal chemistry literature of anywhere between two to nineteen targets for any drug (Bajorath, 2016). An existing drug with this profile might be initially annotated to a particular mechanism and the repurposing effort discovers a new therapeutic indication with a disease mechanism different to that targeted by the starting drug. This type of drug repurposing is sometimes referred to as off-target. The somewhat confusing use of the term “pleiotropy” in the drug context has been described (Hodgkin, 2002; Paaby and Rockman, 2013; Gratten and Visscher, 2016). In repurposing a given drug one might discover a new therapeutic opportunity by way of on-target or off-target effects or possibly both. Metformin is an example of a drug exhibiting both on-target and off-target therapeutic effects (Menendez, 2020).

In drug development it matters whether the new indication for a repurposed drug is on-target or off-target. With newly discovered indications arising from an on-target mechanism, the medicinal chemistry efforts that optimized the compound for the target should effectively have therefore optimized the compound for the new indication, unless perhaps there are significant pK/pD differences in the new indication. Although knowing a drug’s target is not a regulatory requirement there is nonetheless a well-established “comfort level” in the industry with knowing therapeutic mechanism of action.

Conversely, in the case of newly uncovered therapeutic potential that arises from an off-target mechanism, medicinal chemistry efforts used towards the original target will not have optimized the compound for the target of interest for the new indication. Moreover, in these off-target scenarios the target of interest is very often not known. In these instances, computational tools used in medicinal chemistry will not help either because most computational tools are target knowledge based. Often the new disease for which therapy is desired is poorly understood and desired targets are not well-characterized, as is the case in 30 percent of rare or orphan diseases (Roessler et al., 2021). In situations like this the discovery of therapeutic potential arising from off-target effects leaves little opportunity for mechanistic target-based or computational approaches to develop better therapies. An off-target repurposed drug might pejoratively be labelled as “promiscuous” and could carry some of the drug discovery industry’s association of off-target effects with an increased likelihood of toxicity.

To be fair, there is a body of medicinal chemistry literature that describes a drug acting at multiple targets as a desirable “multi-target “drug (Ramsay et al., 2018). Literature also exists that as a chemical compound moves forward in the drug discovery process, the drug generally does not become more selective, rather the number of targets the drug acts on may, often unknowingly, increase as improved activities of the best compound are optimized through the development process (Bajorath, 2016).

## Contribution of genetics to understanding pharmacological pleiotropy

To further explore this question of whether pharmacological pleiotropy is largely an on-target or off-target effect it is useful to step away from pharmacology and review progress that has been made in understanding pleiotropy in the classical genetics sense. The term “pleiotropy was first coined by the German geneticist Ludwig Plate in 1910 (Plate, 1910) although Mendel described the phenomenon earlier, in 1866, without coining a term (Mendel, 1866). Almost 100 years before there was any understanding of the molecular basis of inheritance Mendel noted that the inheritance of seemingly unrelated physical traits sometimes co-segregated as a common gene. In other words that one gene influenced multiple physical traits such as seed color and flower patterns.

Ludwig Plate was a geneticist with a focus on the role of inheritance in evolution. He worked across many organisms but, like Mendel, made the observation that some distinct phenotypes were only explicable through the observation of a single gene. He wrote: “I call a unit of inheritance pleiotropic if several characteristics are dependent upon it; these characteristics will then always appear together and may thus appear correlated” (Plate 1910, quoted from McKusick 1976, *p*. 301) (McKusick, 1976).

Since that time, within the field of genetics, a prominent sub-field has emerged solely focused on an understanding of the nature of pleiotropy. Indeed, giants in the field of genetics such as Sewell Wright, J.B.S Haldane, and Herman Muller have all made contributions to the field of pleiotropy.

Within 20 years of the field developing, a prominent thinker at the time, Sir Ronald Fisher, had developed the theory of universal pleiotropy; the notion that a single mutation can potentially affect all phenotypic traits (Fisher, 1930). At the time this was a serious idea that gained general acceptance. While this prevalent idea faded within 10 years, displaced by the work of Beadle and Tatum in the 1940s and the growing support for their “one gene/one enzyme” hypothesis (Beadle and Tatum, 1941), it is still worthy to note the prominence to which the notion that all genes were pleiotropic had risen.

With the unravelling of the molecular underpinnings of inheritance, beginning with Watson and Crick’s discovery of DNA structure in 1953 (Watson and Crick, 1953), and progressing to DNA sequencing in the 1970s and beyond, we now have a clearer understanding of how one gene can have phenotypic consequences on physiologically diverse processes. Clearly there can be a variety of mechanisms leading to genetic pleiotropy. For example, there is precedent in higher eukaryotes, including humans, to encode multiple protein products through alternative start or stop codons as well as alternative mRNA splicing. These types of mechanisms of pleiotropy are of no relevance to understanding pharmacological pleiotropy in which we are trying to learn about the potential diverse effects of a small molecule modulating a protein target.

However, the molecular age has also uncovered precedent for mutations or deletions for single protein products having multiple physiological consequences with disparate phenotypic effects. These examples of genetic pleiotropy are analogues of drugs modulating a single target and therefore are quite relevant to understanding pharmacological pleiotropy. In more recent years some studies have used genome wide approaches to try to address the proportion of protein products which exhibit pleiotropy and to try to quantitate the number of distinct physiological processes that are affected by individual proteins. Su et al. (2009) surveyed the sequences of 321 genes and their orthologs across a number of vertebrate species to estimate the number of “molecular phenotypes” (physiological functions) associated with each gene (Su et al., 2009). They used precedented mathematical models which quantitated the rate of sequence divergence through evolution as a proxy for the number of physiological processes that a protein was involved with, under the premise that the more processes that a protein is involved with the slower the rate of molecular evolution. By this method the group estimated that the average protein was involved with 6-7 molecular phenotypes.

Interestingly, using an independent and complementary approach, Wagner et al. (2008) also tried to quantitate the number of traits that the average gene effected (Wagner et al., 2008). In this instance, they used strains of backcrossed mice and surveyed their phenotype for 70 skeletal traits that were influenced by 102 quantitative trait loci (QTLs). From this study they determined that a QTL, on average effected 6 traits.

Using a more comprehensive genome wide approach in *Saccharomyces cerevisiae* He and Zhang (2006) evaluated functional genomic data coupled with sequence data and use of the Gene Ontology resource bioinformatic data to determine that most pleiotropic effects are not conferred by multiple molecular functions of a protein but rather multiple molecular consequences of a single molecular function (He and Zhang, 2006).

Collectively, this body of genetic and molecular biology research informs us that many, if not most, proteins effect multiple physiological process perhaps representing different tissues or organ systems. By extension, we should expect that modulating a drug target with a small molecule will influence multiple physiological processes. One of these represents the primary therapeutic effect for which the drug was developed. Other physiological processes of that drug, unrelated to the primary therapeutic effect, may also have therapeutic potential and in this way represent a drug repositioning opportunity. The established prevalence of genetic pleiotropy and the evidence that single gene products customarily effect several physiological processes (Espinosa-Cantú et al., 2020) together provide evidence that we should expect the frequency of drug repositioning opportunity by way of an on-target mechanism to be higher than intuitively predicted.

## MLR-1023 as an example of on-target pleiotropy in a very low MWT drug

**FIGURE 3:**
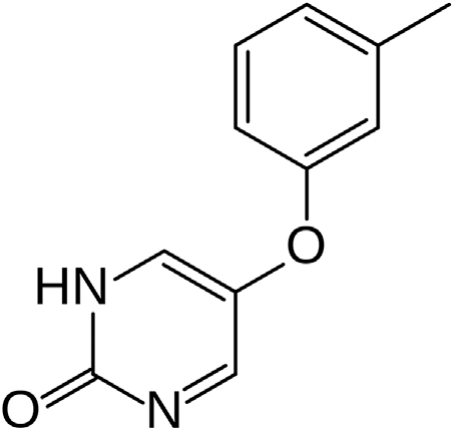
Structure of MLR-1023.

MLR-1023 (Tolimidone; CP-26154; 2(1H)-Pyrimidinone, 5-(3-methylphenoxy; Figure 3) is an interesting example of a drug candidate where the predictions of on-target versus off-target may blur depending upon one’s perspective; biological or chemical. In the biology realm, MLR-1023 is an on-target drug with a novel mechanism. To date, the compound is the only known example of a lyn kinase activator. Various divergent therapeutic effects of MLR-1023 have been described by Melior (Reaume and Lipinski, 2022), by academic sources (Tall and Wang, 2014) and by non-Melior commercial organizations (Ballotti et al., 2020). All the studies investigating mechanism of the various therapeutic actions of MLR-1023 have linked the therapeutic effects to activation of lyn kinase and therefore MLR-1023 exhibits the positive aspects of an on-target repurposed drug.

In the chemistry realm, MLR-1023 is an outlier. Conventional medicinal chemistry precedent would predict that a compound in a low MWT range might be “promiscuous” and more prone to off-target activity because binding affinity for a specific target is typically low and non-specificity is more likely for compounds below MWT 300 (Kozakov et al., 2015; Li, 2020). Yet, the drug exhibits potent *in vitro* activity and high target selectivity making it an outlier for a compound with MWT of 202. Our analysis of the relationship between drug MWT and an index of biological activity across millions of compounds suggests the rarity of a compound like MLR-1023 (Figure 1). Therefore, conventional medicinal chemistry wisdom would predict that the multiple therapeutic activity associated with MLR-1023 arises as an off-target activity. However, experimental investigation into MLR-1023 selectivity and the biology studies associated with MLR-1023 indicate that the compound’s multiple therapeutic activity is the result of a single on-target mechanism. To date, lyn kinase activation is the only mechanistic target discovered for MLR-1023.

Interestingly, consistent with MLR-1023 being an outlier example, its candidacy as a therapeutic candidate was discovered through a discovery strategy that is atypical today but that was common in the 1970s. MLR-1023 was not identified because of a hypothesis-based reasoning predicting the role of lyn kinase as a therapeutic mechanism for a given therapeutic area. Rather, MLR-1023 was originally discovered at Pfizer through an unbiased phenotypic screen in a 1970s era anti-ulcer animal model of human gastrointestinal ulcer disease. Thirty years later the compound was rediscovered by Melior Discovery in a repurposing initiative using a phenotypic screening platform comprising an array of animal models. Speculatively, this suggests that phenotypic assay exploration of the poorly explored region of very low MWT compounds for drug repurposing might be attractive.

MLR-1023 with a lyn kinase EC-50 of 62 nano-molar is a proof-of-principle that low MWT potent drugs can be discovered by phenotypic screening. Proof-of-principle, however, does not equate to proof-of-wide-feasibility. If a phenotypic screen is to be useful the screen throughput must be matched to the screening library characteristics. MLR-1023 was discovered using the an *in vivo* rodent screening platform which is low throughput in an HTS sense. In this instance high value screening substrate (clinically-experienced drug candidates with proven human safety and tolerability) was matched to a lower capacity, but higher predictive quality, *in vivo* rodent screen.

What about phenotypic screening of low MWT, lower chemical value screening substrate? Phenotypic screens in the antibacterial or antiparasitic realm are capable of much higher throughput than *in vivo* rodent screens. Fragment sized libraries have been described that are natural product (NP) inspired with the hope of incorporating desirable NP chemistry features and these libraries when screened for phenotypic antiparasitic activity yield appreciable active subject matter (Pahl et al., 2017; Vu et al., 2018). Future advances in more generalized technology for phenotypic screening on fragment sized low MWT compounds might allow exploration of the largely unexplored biological possibilities for discovery of potent low MWT drugs.

## Conclusion

The era of target-based/mechanism-based drug discovery wherein new drug discovery programs originate with high-throughput screens against a target of interest has created biases in our way of thinking about ideal drug candidates. Prior to this era, drugs below MWT 200 did exist, but attention to *in vitro* target/drug interaction drove a screening-based preference towards higher MWT compounds with higher binding affinities and hopefully, higher specificities. Here we provide evidence that there are drug repositioning opportunities in the very low MWT range and that these low MWT compounds can be expected to be “well-behaved” in the sense that new indications that are uncovered can be on-target effects and not the just the effect of small molecule “promiscuity”. The molecular targets of very low MWT drugs are likely to be difficult to characterize or identify and therefore it is difficult to envision a computational approach to drug repositioning of very small compounds. Nonetheless, phenotypic screening is a proven and effective tool for identifying new therapeutic potential for low MWT repositioning candidates.

## Data Availability

The data analyzed in this study is subject to the following licenses/restrictions: SciFinder-n is a subscription database (https://scifinder-n.cas.org). Requests to access the analyses data should be directed to chris44@aol.com.

## Footnotes

1 Among 2,075,596 compounds only 121,788 (5.868%) had any type of biological study reported.

2 Among 37, 941, 415 compounds 11, 940, 254 (31.470%) were commercially available.
